# The relation of metabolic syndrome according to five definitions to cardiovascular risk factors - a population-based study

**DOI:** 10.1186/1471-2458-9-484

**Published:** 2009-12-23

**Authors:** Cheng-Chieh Lin, Chiu-Shong Liu, Chia-Ing Li, Wen-Yuan Lin, Ming-May Lai, Tsann Lin, Pei-Chia Chang, Yih-Dar Lee, Ching-Chu Chen, Chih-Hsueh Lin, Chuan-Wei Yang, Chih-Yi Hsiao, Walter Chen, Tsai-Chung Li

**Affiliations:** 1 Department of Family Medicine, China Medical University Hospital, Taichung, Taiwan; 2 Department of Family Medicine, College of Medicine, China Medical University, Taichung, Taiwan; 3 Medical Research, China Medical University Hospital, Taichung, Taiwan; 4 Institute of Health Care Administration, College of Health Science, Asia University, Taichung, Taiwan; 5 School and Graduate Institute of Health Care Administration, College of Public Health, China Medical University, Taichung, Taiwan; 6 Administration Center, China Medical University Hospital, Taichung, Taiwan; 7 Lilly Taiwan, Eli Lilly and Company, Taipei, Taiwan; 8 Division of Endocrinology and Metabolism, Department of Medicine, China Medical University Hospital, Taichung, Taiwan; 9 Institute of Health Care Administration, College of Public Health, China Medical University, Taichung, Taiwan; 10 Department of Medicine, China Medical University, Taichung, Taiwan; 11 Institute of Biostatistics, China Medical University, Taichung, Taiwan; 12 Biostatistics Center, China Medical University, Taichung, Taiwan

## Abstract

**Background:**

Although National Cholesterol Education Program (NCEP), International Diabetes Federation (IDF), American Heart Association and National Heart, Lung and Blood Institute (AHA/NHLBI), World Health Organization (WHO), and the European Group for the Study of Insulin Resistance (EGIR) definitions of metabolic syndrome (MetS) have been commonly used by studies, little is known about agreement among these five definitions. We examined the agreement among these five definitions and explored their relationship with risk factors of cardiovascular disease in a Taiwan population.

**Methods:**

A total of 1305 subjects aged 40 years and over in Taiwan were analyzed. Biomedical markers and anthropometric indices were measured. Agreement among definitions was determined by the kappa statistic. Logistic regression models were fit to estimate the odds of a high cardiovascular risk group for five definitions of MetS.

**Results:**

The agreement among the NCEP, IDF, and AHA/NHLBI definitions was from substantial to very good, and agreement between the WHO and EGIR definitions was also substantial. All MetS definitions were significantly associated prevalence of microalbuminuria, elevated highly sensitive CRP (hs-CRP), and arterial stiffness only in women. In men, MetS by NCEP and AHA/NHLBI was associated with elevated level of hs-CRP and arterial stiffness. MetS by WHO and EGIR were significantly associated with microalbuminuria. And MetS by WHO was the only MetS definition that significantly associated with prevalence of arterial stiffness (OR: 2.75, 95% CI: 1.22-6.19).

**Conclusions:**

The associations of these five definitions with cardiovascular risk factors were similar in women, and it was evident that the five definitions performed better in women than in men, with higher ORs observed in relation to arterial stiffness, elevated hs-CRP, and higher Framingham risk scores.

## Background

Metabolic syndrome (MetS) has been shown to increase the risk of CVD mortality and all-cause mortality [1-3]. World Health Organization (WHO) [4] was the first to propose criteria for the diagnosis of MetS, followed by European Group for the Study of Insulin Resistance (EGIR) [5]. In WHO and EGIR definitions, the presence of insulin resistance was a prerequisite. National Cholesterol Education Program (NCEP) Adult Treatment Panel III (ATP-III) assigned MetS as a secondary target for intervention [6]. In 2005, the International Diabetes Federation (IDF) presented a MetS definition [7], in which central obesity was the prerequisite and different cut-off values for waist circumference were introduced for different ethnic groups. The American Heart Association and the National Heart, Lung and Blood Institute (AHA/NHLBI) modified the NCEP criteria by decreasing the glucose cut-off from 110 to 100 mg/dl [8]. Although these definitions have been commonly used by studies, little is known about agreement among these five definitions of MetS, especially the data in the AHA/NHLBI definition.

The value of MetS in health care contexts derives in large part from its potential to reduce the risk of CVD by treating the disease. One way to understand which MetS definitions are more useful in practice is to examine the relationships between these MetS definitions and CVD risk factors. Some factors have been shown to be associated with an increased risk of CVD. Microalbuminuria is one of the strongest predictors of glomerular filtration rate decline, and it is associated with a higher risk of cardiovascular disease and mortality [4,9]. Several cross-sectional studies for Asians had been conducted to examine the relationship of metabolic syndrome with microalbuminuria in senior citizens of rural Japanese [10], in diabetic Japanese patients [11], in urban Chinese residents aged 40 years and over [12], in a Korean adults seeking health check-up [13]. Arterial stiffness and peripheral vascular disease (PVD) are two major underlying pathophysiologies of arterial disease and they are the primary cause of CVD [14]. The effect of MetS defined by NECP, IDF, or AHA/NHLBI criteria on arterial stiffness have been examined in Japanese [15-18], in Korean [19], and in Chinese [20] by cross-sectional studies. Highly sensitive C-reactive protein (hs-CRP) is a marker of systemic inflammation in the body. Mild chronic elevations of hs-CRP concentrations are independently predictive of future cardiovascular events [21,22]. The association of hs-CRP with MetS has been explored in a study of 179 middle-aged Chinese men with a family history of diabetes from a university teaching hospital [23]. The Framingham Risk Scoring System is an index of the 10-year risk of fatal and nonfatal CVD. The effect of the diagnosis of MetS using NECP, WHO and IDF criteria on Framingham risk score have been examined in Korean subjects who seek for physical check-up [24]. The present study seeks to extend existing research by examining the relationship between various MetS definitions and these cardiovascular risk factors simultaneously.

We posed two questions in the present research: what is the estimate of agreement among the various MetS definitions? Which MetS definitions are associated with the risk factors for CVD.

## Methods

### Study population

This was a cross-sectional epidemiological study based on data from the Taichung Community Health Study. A total of 2,359 residents aged 40 and over in Taichung City, Taiwan, participated in October 2004. A two-stage sampling design was used, with a sampling rate proportional to size within each stage. At each stage, simple random sampling was used. In the first stage of sampling, the sampling unit was Li (administrative units) and the selection probability for Li was set at 0.125. A total of 39 Lis were randomly selected from 8 city districts. In the second stage, 110 individuals were randomly selected from each sample Li. A total of 4280 individuals were selected and 750 individuals who were not eligible were excluded. A total of 3,530 subjects were eligible, and 2,359 agreed to participate with an overall response rate of 66.83%. The detailed methodology has been described elsewhere [12,20,25,26].

Insulin levels only were measured in the first 1305 subjects and they were analyzed in the current analysis. This study was approved by the Human Research Committee of China Medical University Hospital. Written informed consent was obtained from each participant.

### Data collection

Anthropometric measurements were obtained from the complete physical examination. Weight and height were measured on an autoanthropometer (super-view, HW-666), with subjects shoeless and wearing light clothing. Body mass index (BMI) was derived from the formula of weight (kg) ÷ (height)^2 ^(m^2^). With the participant standing, waist circumference was measured midway between the superior iliac crest and the costal margin. Percent body fat mass (%FM) was assessed by conventional tetrapolar bioelectrical impedance analysis using the Tanita BC-418 MA Impedanciometer (Tanita Corp., Tokyo, Japan) [27]. Blood pressure was measured using an electronic device (COLIN, VP-1000, Japan).

The measurement of brachial-ankle pulse wave velocity (baPWV) and the ankle-brachial index (ABI) were determined using an automatic waveform analyzer (VP-1000; Colin Co., Komaki, Japan) with well-documented validity and reproducibility (coefficient of variation [CV] = 3.31% and reproducibility coefficient = 0.947). Higher baPWV values indicated more severe arterial stiffness. Lower ABI values indicated more severe PVD. High baPWV was defined as a value higher than 1,400 cm/s, whereas an ABI index <0.9 was considered the presence of PVD [28].

Blood was drawn with minimal trauma from an antecubital vein in the morning, after a 12-hour overnight fasting, and was sent for analysis within four hours of collection. Biochemical markers such as fasting plasma glucose, high-density lipoprotein cholesterol (HDL-C), triglyceride, urine albumin and creatinine were analyzed by a biochemical autoanalyzer (Beckman Coluter Synchron system, Lx-20, Fullerton, CA, USA) at the Clinical Laboratory Department of China Medical University Hospital. Plasma cholesterol and triglyceride levels were determined by an enzymatic colorimetric method. The HDL-C level was measured by a direct HDL-C method and the low-density lipoprotein cholesterol (LDL-C) level was measured by a direct LDL-C method, too. The serum insulin level was measured by a commercial enzyme-linked immunosorbent assay kit (Diagnostic Products, Los Angeles, CA). The interassay CV for insulin was 8.7% and the intra-assay CV was 3.4%. Insulin sensitivity was estimated with a Homeostasis Model Assessment (HOMA-IR) equation. The HOMA-IR equals fasting serum insulin (μU/ml) times fasting plasma glucose (mmol/l) divided by 22.5 [29]. Hs-CRP levels were measured by nephelometry, a latex particle-enhanced immunoassay (TBA-200FR, Tokyo, Japan). The interassay and intraassay CVs were <2.0% and <1.9%, respectively. The lower detection limit of the assay was 0.1 mg/L. The urinary albumin-to-creatinine ratio (ACR) in the morning urine sample was used as a marker of the albumin excretion rate. Urinary creatinine (Jaffe's kinetic method) and albumin (colorimetyl bromcresol purple) were measured by an autoanalyzer. The interassay precision coefficient of variation was <3.0% for both creatinine and albumin concentrations. Urinary ACR ranging from 30 mg g-1 creatinine to 300 mg g-1 creatinine was defined as microalbuminuria [30].

Using the Framingham risk score based on the LDL-C level [31], the estimated total coronary heart disease risk over a 10-year period for every individual was calculated. Data on sociodemographic characteristics, including gender, age, smoking, drinking, physical activity, occupational activity, menopausal status, family history of cardiovascular-related diseases, physician-diagnosed diseases, and medication history were collected when the participants underwent a complete physical examination.

### The metabolic syndrome

Table 1 shows the criteria of the five MetS definitions studied. An Asian modification of the NCEP ATP-III definition of MetS was used [32]. For WHO definition, we used HOMA-IR to define insulin resistance. A similar modification was used in previous studies [33,34]. We defined the subjects in the highest quartile of the HOMA-IR distribution as insulin resistant [5]. The cutoff value of HOMA-IR for non-diabetic subjects was 2.53 in this study and the corresponding cutoff value of EGIR defined insulin resistance was 10.40 μU/ml or 74.6 pmol/L. For medications, they have to be prescribed by their physicians. For hypertension, there are 4 types of treatment: angiotension II converting enzyme inhibitor (ACEI), angiotensis II receptor blocker, calcium channel blocker, and diuretics. For low HDL and raised triglycerides, there are 4 types of treatment: statins, bile acid sequestrants, nicotinic acid, and fibric acids.

**Table 1:** Definitions of metabolic syndrome according to the NCEP ATP III, IDF, AHA, WHO, and EGIR criteria

	Definition of Metabolic Syndrome (MetS)				
	
	NCEP	IDF	AHA	WHO	EGIR
Definition of MetS	Any 3 of 5 criteria listed below	Increased waist plus any of 2 of other 4 criteria	Any 3 of 5 criteria listed below	IFG, IGT, or IR plus 2 of other 5 criteria	Insulin in top 25% plus 2 of other 4 criteria
BMI (kg/m^2^)	--	--	--	>30	--
Abdominal obesity (men/women)	Waist >90/80	Waist ≧90/80	Waist ≧90/80	Waist-to-hip ratio >0.9/0.85	Waist ≧94/80
Triglycerides (nmol/L)	≧1.7 or drug treatment for this lipid abnormality	≧1.7 or drug treatment for this lipid abnormality	≧1.7 or drug treatment for this lipid abnormality	≧1.7 or drug treatment for this lipid abnormality	>2.0 or drug treatment for this lipid abnormality
HDL cholesterol (nmol/L) (men/women)	<1.0/1.3 or drug treatment for this lipid abnormality	<1.0/1.3 or drug treatment for this lipid abnormality	<1.0/1.3 or drug treatment for this lipid abnormality	<0.9/1.0 or drug treatment for this lipid abnormality	<1.0 or drug treatment for this lipid abnormality
Blood pressure (mmHg)	≧130/≧85 or drug treatment for hypertension	≧130/≧85 or drug treatment for hypertension	≧130/≧85 or drug treatment for hypertension	≧140/≧90 or drug treatment for hypertension	≧140/≧90 or drug treatment for hypertension
HOMA-IR	--	--	--	>2.53	--
Fasting glucose (nmol/L)	≧6.1	≧5.6	≧5.6	≧6.1	≧6.1
Fasting insulin (pmol/L)	--	--	--	--	>74.6 (Top 25%)
Urinary albumin excretion	--	--	--	≧30 mg/g creatinine	--


National Cholesterol Education Program (NCEP) Adult Treatment Panel III (ATP-III), International Diabetes Federation (IDF), American Heart Association and National Heart Lung and Blood Institute (AHA/NHLBI), World Health Organization (WHO), European Group for the Study of Insulin Resistance (EGIR), metabolic syndrome (MetS), body mass index (BMI), high-density lipoprotein (HDL), homeostasis model assessment of insulin resistance (HOMA-IR), insulin resistance (IR).

### Statistical analysis

Continuous variables were reported as mean ± standard deviation (SD) and categorical variables were reported as percentages. Agreement between the five definitions of MetS was determined using the kappa statistic (κ). The level of agreement was considered poor with 0.20, fair with κ = 0.21-0.40, moderate with κ = 0.41-0.60, substantial with κ = 0.61-0.80, and very good with κ > 0.80 [35]. Logistic regression models were fit to estimate the odds of high cardiovascular risk group for five definitions of MetS. The high risk groups for %FM, hs-CRP, and Framingham risk scores were determined by the upper quartile of their distribution. All reported p values were those of two-sided tests; statistical significance was set at p < 0.05. All analyses were performed using SAS version 9.1 (SAS Institute Inc, Cary, NC).

## Results

The distributions of sociodemographic, anthropometric and biochemical characteristics and prevalence of metabolic syndrome according to 5 definitions for all study subjects, and according to gender are summarized in Table 2. Agreement among the five definitions of MetS is shown in Table 3. Agreement among the NCEP ATP-III, IDF, and AHA/NHLBI definitions was from substantial to very good (κ: 0.63-0.84 for men and 0.68-0.85 for women), and that between the WHO and EGIR definitions was also substantial (κ: 0.64 for men and 0.65 for women). Agreement between NCEP ATP-III, IDF or AHA/NHLBI and WHO or EGIR definitions was from fair to moderate for both men and women.

**Table 2:** Distributions of sociodemographic, anthropometric and biochemical characteristics, and prevalence of metabolic syndrome according to 5 definitions for all study subjects, and according to gender

	Mean (SD)		
	**Overall** **(N = 1305)**	**Men** **(N = 633)**	**Women** **(N = 672)**

Age (years)	55.99 (11.25)	57.71 (12.23)	54.37 (9.98)
Smoking (%)^†^	198 (15.18)	175 (27.65)	23 (3.43)
Drinking (%)^†^	314 (24.08)	242 (38.23)	72 (10.73)
Betel nut chewing (%)^†^	40 (3.07)	39 (6.18)	1 (0.15)
Exercise (%)^†a^	865 (66.33)	423 (66.93)	442 (65.77)
Body mass index (kg/m^2^)	24.35 (3.26)	24.87 (3.11)	23.85 (3.33)
Waist circumference (cm)	81.71 (9.87)	86.74 (8.55)	76.98 (3.61)
Waist-to-hip ratio	0.85 (0.07)	0.89 (0.05)	0.81 (0.06)
Fasting blood glucose (mmol/l)	5.74 (1.53)	5.87 (1.53)	5.61 (1.53)
Fasting insulin (pmol/l)	60.92 (48.50)	64.65 (48.38)	57.40 (48.43)
HOMA-IR	2.06 (2.23)	2.43 (2.13)	2.11 (2.30)
Triglyceride (mmol/l)	1.32 (0.99)	1.49 (1.16)	1.16 (0.76)
HDL-cholesterol (mmol/l)	1.20 (0.34)	1.07 (0.28)	1.31 (0.34)
LDL-cholesterol (mmol/l)	3.34 (0.88)	3.37 (0.87)	3.32 (0.89)
Diastolic blood glucose (mmHg)	78.37 (11.94)	81.77 (10.82)	75.14 (12.05)
Systolic blood glucose (mmHg)	133.68 (20.68)	136.51 (19.53)	131.02 (21.38)
Hypertension (%)^†^	354 (27.21)	189 (29.91)	165 (24.66)
Family history of diabetes (%)^†^	330 (25.10)	157 (24.84)	173 (25.74)
Microalbuminuria (%/ACR ≧30 μg/min)^†^	278 (21.38)	120 (18.99)	158 (23.65)
PVD^†b^	86 (6.59)	37 (5.85)	49 (7.29)
Arterial stiffness^†c^	838 (64.76)	455 (72.45)	383 (57.51)
%FM	31.59 (7.62)	26.45 (5.50)	36.40 (6.02)
Hs-CRP (mg/L)	0.25 (0.50)	0.26 (0.54)	0.23 (0.46)
Metabolic syndrome by NCEP ATP-III	410 (31.42)	226 (35.70)	184 (27.38)
Metabolic syndrome by IDF	328 (25.13)	167 (26.38)	161 (23.96)
Metabolic syndrome by AHA	501 (38.39)	275 (43.44)	226 (33.63)
Metabolic syndrome by WHO	246 (18.85)	154 (24.33)	92 (13.69)
Metabolic syndrome by EGIR*	203 (15.56)	123 (19.43)	80 (11.90)


homeostasis model assessment of insulin resistance (HOMA-IR), high-density lipoprotein (HDL), low-density lipoprotein (LDL), albumin-creatinine ratio (ACR), ankle-brachial index (ABI), brachial-ankle pulse wave velocity (baPWV), percent body fat mass (%FM), peripheral vascular disease (PVD), highly sensitive C-reactive protein (hs-CRP), National Cholesterol Education Program (NCEP) Adult Treatment Panel III (ATP-III), International Diabetes Federation (IDF), American Heart Association and National Heart Lung and Blood Institute (AHA/NHLBI), World Health Organization (WHO), European Group for the Study of Insulin Resistance (EGIR). ^†^: n (%). *:A total of 1305 samples, includes 633 men and 672 women. a: Participants reported they spent at least 20 minutes in any types of recreational activity at least 3 times per week for more than 6 months. b: peripheral vascular disease (PVD): ankle-brachial index (ABI) < 0.9. c: arterial stiffness: brachial-ankle pulse wave velocity (baPWV) > 1400 cm/s.

**Table 3:** Agreement between each definition of the metabolic syndrome.^a^

	NCEP	IDF	AHA	WHO
*Men*				
IDF	0.63 (0.57-0.70)			
AHA	0.84 (0.80-0.88)	0.64 (0.58-0.69)		
WHO	0.49 (0.42-0.56)	0.40 (0.32-0.48)	0.43 (0.36-0.49)	
EGIR	0.44 (0.37-0.52)	0.47 (0.39-0.55)	0.37 (0.31-0.44)	0.64 (0.56-0.71)
*Women*				
IDF	0.68 (0.62-0.75)			
AHA	0.85 (0.81-0.90)	0.77 (0.71-0.82)		
WHO	0.49 (0.42-0.57)	0.43 (0.35-0.51)	0.43 (0.36-0.50)	
EGIR	0.40 (0.32-0.48)	0.40 (0.32-0.49)	0.37 (0.30-0.43)	0.65 (0.57-0.74)

a: kappa statistics and their 95% confidence interval were presented.

National Cholesterol Education Program (NCEP) Adult Treatment Panel III (ATP-III), International Diabetes Federation (IDF), American Heart Association and National Heart Lung and Blood Institute (AHA/NHLBI), World Health Organization (WHO), European Group for the Study of Insulin Resistance (EGIR).

The adjusted odds ratios (ORs) of the high risk group for cardiovascular risk factors according to each definition of MetS are shown in Table 4. All MetS definitions were significantly associated with prevalence of high %FM and high Framingham risk scores in both men and women. In men, MetS by NCEP, AHA and WHO were associated with an increase prevalence of arterial stiffness and hs-CRP. In addition, MetS by NCEP, AHA, WHO and EGIR were significantly associated with microalbuminuria, and MetS by the WHO was the only MetS definition that significantly associated with PVD. In women, all MetS definitions were significantly associated with a prevalence of microalbuminuria, arterial stiffness, higher % FM, and elevated hs-CRP, except for the EGIR with microalbuminuria. In men, MetS of all definitions had larger ORs with high %FM than with the other CVD risk factors, while in women, MetS had larger ORs with high %FM and Framingham risk scores than with the other CVD risk factors.

**Table 4:** The adjusted OR for metabolic syndrome of each studied definition associated with cardiovascular risk factors

	**Adjusted OR (95% CI)^a^**									
	
	**Men (N = 633)**					**Women (N = 672)**				
		
	**NCEP**	**IDF**	**AHA**	**WHO**	**EGIR**	**NCEP**	**IDF**	**AHA**	**WHO**	**EGIR**
	
Microalbuminuria	1.99** (1.31-3.03)	1.51 (0.97-2.35)	1.64* (1.08-2.50)	4.44*** (2.85-6.91)	2.62*** (1.49-4.60)	2.21*** (1.47-3.32)	2.29*** (1.51-3.48)	2.26*** (1.52-3.38)	4.16*** (2.57-6.73)	1.80 (0.99-3.29)
PVD	1.29 (0.65-2.59)	1.48 (0.72-3.20)	1.36 (0.69-2.70)	2.24* (1.11-4.55)	2.17 (0.92-5.10)	1.68 (0.83-3.40)	1.20 (0.57-2.54)	1.17 (0.58-2.33)	1.34 (0.56-3.19)	1.12 (0.38-3.33)
Arterial stiffness	3.07*** (1.92-4.92)	1.27 (0.79-2.03)	2.90*** (1.88-4.48)	1.95* (1.15-3.29)	1.40 (0.80-2.47)	3.72*** (2.23-6.20)	3.18*** (1.88-5.40)	3.99*** (2.50-6.35)	6.61*** (2.93-14.92)	4.63** (2.02-10.63)
Higher %FM	6.93*** (4.66-10.28)	11.29*** (7.39-17.23)	5.55*** (3.73-8.26)	5.58*** (3.72-8.37)	10.76*** (6.35-18.23)	4.56*** (3.05-6.82)	8.88*** (5.78-13.65)	4.31*** (2.90-6.42)	3.24*** (2.01-5.23)	8.30*** (4.47-15.41)
Elevated hs-CRP	1.78** (1.23-2.59)	1.15 (0.77-1.73)	1.71** (1.19-2.48)	1.66* (1.11-2.50)	1.45 (0.87-2.42)	3.09*** (2.07-4.62)	2.25** (1.49-3.39)	2.63***(1.78-3.89)	4.42*** (2.73-7.15)	4.14*** (2.28-7.52)
Higher risk scores^b^	4.13*** (2.62-6.51)	2.60*** (1.64-4.13)	3.82***(2.44-5.98)	4.65*** (2.84-7.62)	2.79*** (1.48-5.26)	9.51*** (5.94-15.21)	4.77*** (3.00-7.59)	10.25*** (6.54-16.07)	10.57*** (5.48-20.42)	5.54*** (2.73-11.27)

^a^Logistic regression adjusted for age, smoking, alcohol drinking, betel nut chewing, low income, education.

^b^Framingham risk score^31 ^; microalbuminuria: albumin-creatinine ratio (ACR) >30 mg g-1 creatinine, peripheral vascular disease (PVD): ankle-brachial index (ABI) <0.9; arterial stiffness: brachial-ankle pulse wave velocity (baPWV) >1400 cm/s; higher percent body fat mass (%FM) greater than the cutoff value of 3^th ^quartile; elevated highly sensitive C-reactive protein (hs-CRP): greater than the cutoff value of 3^th ^quartile; higher risk scores: Framingham risk score greater than the cutoff value of 3^th ^quartile.

*: p < 0.05; **: p < 0.01; ***: p < 0.001

In additional file 1 Table S1 shows the relationships between cardiovascular risk factors and components of metabolic syndrome according to five definitions. In men, most of components were associated with significant adjusted OR in a similar way with the exception of obesity component of WHO with arterial stiffness, obesity component of EGIR with elevated hs-CRP, raised TG component of NCEP/IDF/AHA/WHO with elevated hs-CRP, raised blood pressure of NCEP/IDF/AHA with PVD, raised fasting glucose of IDF/AHA and WHO with elevated hs-CRP. In women, most of components were also associated with significant adjusted OR in a similar way with the exception of obesity component of NCEP, IDF/AHA/EGIR with arterial stiffness, raised TG component of EGIR with PVD and higher %FM, low HDL-cholesterol of NCEP/IDF/AHA with arterial stiffness and higher %FM, raised fasting glucose of NCEP/EGIR and WHO with higher %FM and elevated hs-CRP.

## Discussion

Our data show the kappa agreement between the definitions not considering insulin measurement (NCEP, AHA/NHLBI, or IDF) and the definitions requiring insulin measurement (WHO or EGIR) ranged from 0.33 to 0.46 in men and from 0.34 to 0.42 in women, and was from fair to moderate, similar to previous findings [34,36]. AHA/NHLBI was not evaluated in Can's and Dekker's work and IDF was not evaluated in Dekker's work. But the agreement between IDF and WHO definitions in our study (κ = 0.37) was much lower than in Guerrero-Romero's work (κ = 0.51) [37]. We found a substantial agreement (κ = 0.67) between NCEP and IDF definitions, much lower than those in Can's and Guerrero-Romero's studies (both κ = 0.87), but higher than that (κ = 0.54) in the Korean Health and Examination Survey [38].

Our study identified similarities among the five definitions of MetS, but also revealed differences. In men, all definitions were associated with high %FM and high Framingham risk scores. The only definitions associated with microalbuminuria were those considering insulin resistance (WHO and EGIR criteria), and WHO definition was the only predictor of PVD. On the other hand, the NCEP ATP III and AHA definitions, which did not consider insulin resistance, were associated with arterial stiffness and elevated hs-CRP. IDF definition was not correlated with arterial stiffness or elevated hs-CRP, possibly due to its prerequisite factor. In general, the associations of these five definitions with cardiovascular risk factors were similar in women, and it was evident that the five definitions in women performed better than in men, with higher ORs observed in relation to arterial stiffness, elevated hs-CRP, and higher Framingham risk scores, and more ORs significantly related to microalbuminuria.

The reason why IDF definition correlated less well with arterial stiffness or elevated hs-CRP, compared to NCEP ATP III or AHA/NHLBI definition, may be that central obesity is a prerequisite factor of IDF definition. Central obesity is believed to be associated with insulin resistance and has been suggested to induce insulin resistance and hyperinsulinemia, owing to the influence of free fatty acids, derived from visceral fat, on the liver [39]. Khoo et al. [40] determined the effect of the presence of central obesity on insulin resistance and other cardiovascular risk factors. They found that central obesity is important for the identification of individuals with insulin resistance and glucose intolerance; whereas AHA/NHLBI definition is more appropriate in identifying those at increased risk of cardiovascular disease. A significant proportion of individuals in their population (ranging from 10% to 13% in men and 2% to 4% in women) exhibited multiple features of MetS in the absence of central obesity. In our study, this proportion was even higher (14.75% in men and 7.99% in women). These individuals would be diagnosed as having MetS according to NCEP ATP III or AHA/NHLBI criteria, but not by IDF criteria. Thus, MetS according to IDF criteria has lower prevalence. Under such condition, IDF definition will have less power, which may explain why IDF definition was less well correlated with cardiovascular risk factors in our study and previous work.

One of the limitations of our study is that it was cross-sectional and examined cardiovascular risk factors among MetS definitions. Although many studies have compared different definitions [34,36,38,41-50], only one has compared more than four published definitions of MetS [36]. Instead of including the NCEP definition, Can's work examined the American College of Endocrinology (ACE) definition. In addition, microalbuminuria was not counted as a component in the WHO definition in their study. Although our study only provided cross-sectional relationships between MetS and cardiovascular risk factors, no cross-sectional study has compared five published definitions in relation to hs-CRP, arteriosclerosis, and PVD. Another limitation of our study is that insulin sensitivity was determined by HOMA-IR, and not the insulin sensitivity index derived from the hyperinsulinemic euglycemic clamp. Previous studies indicated that there existed a moderate to strong relationship between the HOMA-IR value and the insulin sensitivity index [51,52]. However, the insulin sensitivity index is not feasible for epidemiologic studies involving large numbers of participants. In addition, we did not perform oral glucose tolerance testing, so we may not provide valid estimate for the prevalence of MetS for the EGIR and WHO definitions by included some patients with T2DM or excluding some hyperglycemic cases that could be detected by glucose tolerance testing.

## Conclusions

In conclusion, among the NCEP-ATP III, IDF, and AHA/NHLBI definitions of MetS, the agreement among the definitions that do not require the measurement of insulin levels are from substantial to very good agreement, and the agreement between the WHO and EGIR definitions, the definitions that require the measurement of insulin sensitivity and fasting insulin levels, is substantial. Our data suggest that different definitions of MetS were correlated with all cardiovascular risk factors in women, but with different cardiovascular risk factors in men. In men, the modified NCEP ATP III and AHA/NHLBI (but not IDF) definitions were associated with an elevated level of hs-CRP and arterial stiffness independently of high-risk lifestyle behaviors, but the WHO definition seems to be more related to microalbuminuria and PVD. Since serum insulin, hs-CRP, baPWV, ABI, and urinary albumin are relatively costly measures compared with the other routine biologic markers, they are not feasible to be collected in everyday practice. Thus, our study findings provided new insight for diagnosis of MetS using various definitions in clinical practice.

## Abbreviations

CVD: cardiovascular disease; HDL-C: high-density lipoprotein cholesterol; Mets: metabolic syndrome; WHO: World Health Organization; EGIR: European Group for the Study of Insulin Resistance; NCEP/ATP-III: National Cholesterol Education Program/Adult Treatment Panel III; AHA/NHLBI: American Heart Association and the National Heart, Lung and Blood Institute; IDF: International Diabetes Federation; hs-CRP: Highly sensitive C-reactive protein; baPWV: brachial-ankle pulse wave velocity; ABI: ankle-brachial index; LDL-C: low-density lipoprotein cholesterol; HOMA-IR: insulin sensitivity was estimated with a Homeostasis Model Assessment; ACR: urinary albumin-to-creatinine ratio.

## Competing interests

The authors declare that they have no competing interests.

## Authors' contributions

CCL and TCL contributed equally to the design of the study and direction of its implementation, including supervision of the field activities, quality assurance and control. CSL, CIL, WYL, CCC, MML, TL, CYH, WC and PCC supervise the field activities. CSL and YDL helped conduct the literature review and prepare the Methods and the Discussion sections of the text. TCL, CSL, CCL, CHL, CWY and CIL designed the study's analytic strategy and conducted the data analysis. All authors read and approved the final manuscript.

## Pre-publication history

The pre-publication history for this paper can be accessed here:

http://www.biomedcentral.com/1471-2458/9/484/prepub

## Supplementary Material

Additional file 1 **Table S1**. The relationships between cardiovascular risk factors and components of metabolic syndrome according to five definitions. Click here for file
