# Supplementary material for: The relation of metabolic syndrome according to five definitions to cardiovascular risk factors - a population-based study
Source: BMC Public Health. 2009 Dec 23;9:484. doi: 10.1186/1471-2458-9-484 (PMC2805641; doi:10.1186/1471-2458-9-484)
Supplement: Additional file 1 — Table S1. The relationships between cardiovascular risk factors and components of metabolic syndrome according to five definitions. [file 1471-2458-9-484-S1.DOC]

Table S1 The adjusted OR for metabolic syndrome of each studied definition associated with cardiovascular risk factors

|  | Microalbuminuria | ABI | baPWV | Higher %FM | Elevated hs-CRP | Higher risk scores^b^ |
| --- | --- | --- | --- | --- | --- | --- |
| ***Male*** |  |  |  |  |  |  |
| Obesity |  |  |  |  |  |  |
| NCEP | 1.74  (1.13-2.66)* | 1.08  (0.52-2.22) | 1.32  (0.84-2.06) | 19.19  (12.27-30.02)*** | 1.28  (0.87-1.89) | 2.03  (1.29-3.19)** |
| IDF/AHA | 1.62  (1.06-2.64)* | 1.52  (0.77-3.00) | 1.18  (0.77-1.82) | 18.70  (11.92-29.33)*** | 1.26  (0.86-1.84) | 2.25  (1.45-3.51)*** |
| WHO | 1.62  (1.07-2.47)* | 1.91  (0.95-3.83) | 1.61  (1.06-2.44)* | 6.09  (4.06-9.13)*** | 1.20  (0.83-1.74) | 2.72  (1.75-4.20)*** |
| EGIR | 2.05  (1.28-3.29)** | 1.78  (0.85-3.74) | 1.32  (0.79-2.21) | 17.04  (10.46-27.76)*** | 1.61  (1.04-2.49)* | 1.92  (1.14-3.23)* |
| Triglycerides |  |  |  |  |  |  |
| NCEP/IDF/AHA/WHO | 1.72  (1.12-2.65)* | 1.36  (0.68-2.72) | 2.50  (1.59-3.94)*** | 2.49  (1.71-3.63)*** | 2.12  (1.45-3.11)*** | 3.24  (20.2-5.20)*** |
| EGIR | 1.81  (1.10-2.96)* | 0.4  (0.14-1.17) | 1.86  (1.11-3.11)* | 2.03  (1.33-3.12)** | 1.41  (0.90-2.20) | 2.90  (1.71-4.93)*** |
| HDL |  |  |  |  |  |  |
| NCEP/IDF/  AHA/EGIR | 1.13  (0.74-1.72) | 0.93  (0.47-1.84) | 0.96  (0.64-1.43) | 1.76  (1.21-2.55)** | 1.25  (0.86-1.80) | 1.66  (1.07-2.58)* |
| WHO | 1.28  (0.83-1.97) | 1.37  (0.68-2.77) | 1.11  (0.72-1.73) | 1.78  (1.22-2.59)** | 1.20  (0.82-1.77) | 1.18  (1.10-2.67)* |
| Blood pressure |  |  |  |  |  |  |
| NCEP/IDF/AHA | 2.40  (1.55-3.71)*** | 2.52  (1.23-5.18)* | 7.61  (4.58-12.63)*** | 3.37  (2.30-4.96)*** | 1.63  (1.12-2.37)* | 2.95  (1.90-4.58)*** |
| WHO/EGIR | 2.48  (1.62-3.80)*** | 1.96  (0.97-3.98) | 9.49  (4.73-19.04)*** | 3.16  (2.14-4.65)*** | 1.69  (1.14-2.49)** | 3.73  (2.39-5.85)*** |
| Fasting glucose |  |  |  |  |  |  |
| NCEP/ EGIR | 2.56  (1.64-4.00)*** | 1.11  (0.50-2.46) | 2.23  (1.27-3.94)** | 2.13  (1.42-3.21)*** | 1.50  (0.98-2.28) | 4.95  (2.98-8.23)*** |
| IDF/AHA | 1.69  (1.11-2.58)* | 0.64  (0.31-1.33) | 1.58  (1.04-2.39)* | 1.69  (1.17-2.43)** | 1.49  (1.03-2.16)* | 3.52  (2.25-5.52)*** |
| WHO | 2.26  (1.48-3.44)*** | 1.56 (0.79-3.06) | 2.31  (1.51-3.54)*** | 4.43  (3.02-6.49)*** | 1.70  (1.17-2.45)** | 4.48  (2.81-7.15)*** |
| ***Female*** |  |  |  |  |  |  |
| Obesity |  |  |  |  |  |  |
| NCEP | 2.00  (1.35-2.96)*** | 0.94  (0.47-1.89) | 1.60  (1.03-2.48)* | 16.79  (10.83-26.03)*** | 2.77  (1.89-4.07)*** | 2.39  (1.57-3.65)*** |
| IDF/AHA  EGIR | 2.02  (1.38-2.98)*** | 1.03  (0.53-2.00) | 1.65  (1.09-2.50)* | 22.92  (14.19-37.01)*** | 2.51  (1.72-3.65)*** | 2.73  (1.81-4.10)*** |
| WHO | 1.59  (1.06-2.40)* | 1.36  (0.69-2.70) | 1.59  (1.00-2.54) | 4.28  (2.88-6.37)*** | 2.39  (1.61-3.53)*** | 2.78  (1.78-4.35)*** |
| Triglycerides |  |  |  |  |  |  |
| NCEP/IDF/AHA/WHO | 1.34  (0.86-2.08) | 1.79  (0.90-3.55) | 1.77  (1.08-2.90)* | 1.40  (0.91-2.15) | 2.36  (1.56-3.59)*** | 4.06  (2.50-6.60)*** |
| EGIR | 1.63  (0.92-2.91) | 2.46  (1.07-5.64)* | 2.33  (1.13-4.80)* | 2.38  (1.36-4.16)** | 2.91  (1.68-5.06)*** | 6.22  (3.15-12.29)*** |
| HDL |  |  |  |  |  |  |
| NCEP/IDF/ AHA | 1.37  (0.94-2.01) | 0.82  (0.45-1.49) | 1.47  (1.00-2.14)* | 1.94  (1.33-2.83)*** | 1.84  (1.27-2.66)** | 7.18  (4.41-11.68)*** |
| WHO/ EGIR | 1.09  (0.72-1.67) | 0.51  (0.22-1.18) | 1.48  (0.94-2.33) | 1.26  (0.84-1.89) | 1.59  (1.07-2.36)* | 2.55  (1.63-4.00)*** |
| Blood pressure |  |  |  |  |  |  |
| NCEP/IDF/AHA | 2.52  (1.69-3.76)*** | 1.23  (0.61-2.46) | 5.63  (3.37-9.42)*** | 2.22  (1.50-3.27)*** | 1.72  (1.16-2.54)** | 4.22  (2.76-6.44)*** |
| WHO/EGIR | 2.97  (1.95-4.51)*** | 0.90  (0.41-2.00) | 6.30  (3.41-11.64)*** | 2.24  (1.49-3.37)*** | 1.94  (1.28-2.94)** | 3.77  (2.40-5.94)*** |
| Fasting glucose |  |  |  |  |  |  |
| NCEP/ EGIR | 2.57  (1.60-4.12)*** | 1.05  (0.41-2.68) | 2.32  (1.20-4.49)* | 1.65  (1.03-2.65)* | 2.17  (1.36-3.47)** | 4.22  (2.40-7.43)*** |
| IDF/AHA | 2.01  (1.36-2.98)*** | 0.82  (0.40-1.67) | 2.57  (1.64-4.04)*** | 1.46  (0.99-2.45) | 1.44  (0.98-2.12) | 2.77  (1.81-4.21)*** |
| WHO | 1.85  (1.26-2.72)** | 1.08  (0.55-2.10) | 2.13  (1.38-3.27)*** | 2.92  (2.00-4.26)*** | 2.99  (2.06-4.36)*** | 4.54  (2.95-7.00)*** |

^a^Logistic regression adjusted for age, smoking, alcohol drinking, betel nut chewing, low income, education. ^b^Framingham Risk Score. ^29^

Percent body fat mass (%FM), highly sensitive C-reactive protein (hs-CRP); *: p<0.05; **: p<0.01; ***: p<0.001.
